# Assessing the genetic integrity of sugarcane germplasm in the USDA-ARS National Plant Germplasm System collection using single-dose SNP markers

**DOI:** 10.3389/fpls.2023.1337736

**Published:** 2024-01-04

**Authors:** Sunchung Park, Dapeng Zhang, Gul Shad Ali

**Affiliations:** ^1^ Sustainable Perennial Crops Laboratory, United States Department of Agriculture, Agriculture Research Service, Beltsville, MD, United States; ^2^ Subtropical Horticulture Research Station, United States Department of Agriculture, Agriculture Research Service, Miami, FL, United States

**Keywords:** *Saccharum*, germplasm, sugarcane, genetic diversity, population structure

## Abstract

The World Collection of Sugarcane and Related Grasses, maintained at the USDA-ARS in Miami, FL, is one of the largest sugarcane germplasm repositories in the world. However, the genetic integrity of the *Saccharum* spp. germplasm in this collection has not been fully analyzed. In this study, we employed a single-dose SNP panel to genotype 901 sugarcane accessions, representing six *Saccharum* species and various hybrids. Our analysis uncovered a high rate of clone mislabeling in the collection. Specifically, we identified 86 groups of duplicates, characterized by identical SNP genotypes, which encompassed 211 accessions (23% of the total clones), while 135 groups, constituting 471 clones (52% of the total), exhibited near-identical genotypes. In addition, twenty-seven homonymous groups were detected, which shared the same clone name but differed in SNP genotypes. Hierarchical analysis of population structure partitioned the *Saccharum* germplasm into five clusters, corresponding to *S. barberi, S. sinense, S. officinarum, S. spontaneum* and *S. robustum/S. edule*. An assignment test, based on the five *Saccharum* species, enabled correcting 141 instances of mislabeled species memberships and inaccuracies. Moreover, we clarified the species membership and parentage of 298 clones that had ambiguous passport records (e.g., ‘*Saccharum spp*’, ‘unknown’, and ‘hybrid’). Population structure and genetic diversity in these five species were further supported by Principal Coordinate Analysis and neighbor-joining clustering analysis. Analysis of Molecular Variance revealed that within-species genetic variations accounted for 85% of the total molecular variance, with the remaining 15% attributed to among-species genetic variations. The single-dose SNP markers developed in this study offer a robust tool for characterizing sugarcane germplasm worldwide. These findings have important implications for sugarcane genebank management, germplasm exchange, and crop genetic improvement.

## 1 Introduction

Sugarcane (*Saccharum* spp.) is a prolific energy crop that serves as a substantial source of sugar, biofuel, and other industrial chemicals (Formann et al., 2020). It is cultivated worldwide in over 95 countries across 26.4 million hectares, with a total production of 1.86 billion metric tons and a gross production value of $96.5 billion dollars (FAO, 2023). Sugarcane plays a crucial role in the economies of many tropical and subtropical countries, meeting nearly 80% of global sugar demands for food consumption and accounting for approximately 40% of the world’s bioethanol needs (Lam et al., 2009). Furthermore, lignocellulosic biomass derived from sugarcane and energy cane is recognized as a promising feedstock for biofuel production. As the demand for sugar and biofuel continues to rise, a challenge for sugarcane breeding programs is to develop improved varieties with higher yield, sucrose content, disease resistance, improved ratooning ability and adaptability to environmental stresses. Genetic diversity is crucial in developing such varieties to unlock the full potential of sugarcane as a feedstock for both sugar and fiber production. (Lam et al., 2009; Hoang et al., 2015).Sugarcane species belong to the grass family *Poacea*, genus *Saccharum*, and share genetic similarities with *Sorghum* and other grasses (Spangler et al., 1999). Within the *Saccharum* genus, six main species are recognized: two wild species, *S. spontaneum* (2*n* = 40 -128, *x* = 8) and *S. robustum* (2*n* = 60 – 80), and four cultivated species, *S. officinarum* (2*n* = 80, *x* =10), *S. barberi* (2*n* = 81 -124), *S. sinense* (2*n* = 111 – 120), and *S. edule* (2*n* = 60, 70, 80) (Moore et al., 2013). Genetic studies suggest that *S. officinarum* and *S. edule* originated from *S. robustum* (Grivet et al., 2004; Grivet et al., 2006) and that *S. sinense* and *S. barberi* are interspecific hybrids resulting from a cross between *S. officinarum* an *S. spontaneum*, with 32 -39% of their genomes derived from *S. spontaneum* (D’Hont et al., 1996; Piperidis et al., 2010). Most modern sugarcane cultivars are complex polyploids (*2n = 4x* to 12*x*, totaling 100 – 128 chromosomes), resulting from interspecific crosses between sugar-rich *S. officinarum* and *S. spontaneum* with disease resistance, vigor and other agronomic traits (Piperidis et al., 2010).

Since the early 1970s, sugarcane productivity has steadily increased, largely attributed to improved varieties and agronomic practices (Moore et al., 2013; Hale et al., 2022). However, the sugarcane industry faces challenges posed by diseases, pests, adaptability to different soil types, water availability and environmental stresses, underscoring the need for the development of new and resilient sugarcane varieties with high sucrose content. The current repertoire of modern sugarcane cultivars is the result of crosses made in the early 1900s, involving fewer than 20 *S. officinarum* and *S. spontaneum* clones as parents (Deren, 1995; Raboin et al., 2006), essentially resulting in a monoculture of a few dominant sugarcane varieties grown across large geographic areas, and making them vulnerable to disease and pest outbreaks. To enhance the resilience of sugarcane varieties, it is important to increase the genetic diversity of sugarcane germplasm. Recognizing the importance of landraces and wild relatives as sources of novel genetic traits, efforts for enhancing genetic diversity should be focused on introgression of genes from landraces, wild species within *Saccharum* species complex, and crossable wild relatives such as *Miscanthus* and *Tripidium*. The World Collection of Sugarcanes and Related Grasses (WCSRG), maintained by the United States Department of Agriculture (USDA), Agriculture Research Service (ARS), Subtropical Horticulture Research Station (SHRS), serves as a repository for one of the world largest collections of sugarcane and its wild relatives, originating from various geographical regions. This collection has been used in breeding programs and biological studies to improve sugarcane varieties (Nayak et al., 2014; Zhang et al., 2018; You et al., 2019; Fickett et al., 2020; Hale et al., 2022; Wang et al., 2022). Moreover, as a USDA National Plant Germplasm collection, the clones are freely distributed to international sugarcane community. From 2010 to 2021, a total of 9439 cuttings of various *Saccharum* spp., and 298 cuttings of 4 *Tripidium* spp., were distributed to researchers and breeders in the USA (44%) and internationally (56%) (Hemaprabha et al., 2022).

The WCSRG currently houses approximately 1164 accessions, the majority of which belong to *Saccharum* spp, including 307 *S. spontaneum* accessions, 158 *S. officinarum* accessions, 127 *S. hybrid* accessions, 81 *S. robustum* accessions, 48 *S. sinense* accessions, and 33 *S. barberi* accessions. These accessions originated from various geoclimatic regions and likely harbor genes for adaptation to different climatic stresses, pests, and diseases. Ample information on genetic diversity and population structure within this collection has been generated using molecular markers (Brown et al., 2007; Nayak et al., 2014; Fickett et al., 2020; Xiong et al., 2022) and candidate genes (Parco et al., 2017). Based on simple sequence repeat (SSR) genotyping results, a core collection including 300 accessions was proposed (Nayak et al., 2014). Furthermore, target enrichment sequencing was performed on 307 germplasm accessions from this collection, leading to the identification of ancestor of ancient and modern hybrids in *Saccharum* spp. (Yang et al., 2019). Based on the sequencing data, a genome-wide association study was performed on this diversity panel and candidate genes for agronomic traits and disease resistance were identified (Yang et al., 2019; Yang et al., 2020).

Despite the progress achieved in the molecular characterization of the WCSRG, genetic integrity of the sugarcane germplasm maintained in this collection has not been systematically analyzed. This is mainly because accurate identification of sugarcane germplasm has been technically challenging, due to the high polyploidy (and aneuploidy) nature of this crop (Brown et al., 2007; Song et al., 2016). For any given locus in sugarcane, there can be 8 to 12 alleles in different configurations, which ambiguates genotype identification. Therefore, single-dose molecular markers are needed to distinguish among sugarcane genotypes with complex allele configurations (Song et al., 2016). Recently, You et al. (2019) reported the target enrichment sequencing of 300 sugarcane accessions selected from the world collection and developed an Affymetrix Axiom 100K SNP array. This array, which comprises 31,449 single-dose SNPs and 68,648 low-dosage SNPs, provides a powerful tool for using single-dose SNPs in sugarcane germplasm identification.

In this study, we selected 2000 single-dose SNP markers from the Affymetrix Axiom SNP array (You et al., 2019). After validating the selected candidate SNPs in a pilot study, we selected the final genotyping panel and used it to genotype all the *Saccharum* germplasm, including six *Saccharum* species and hybrids. Through comprehensive genotyping and population structure analyses, we assessed genetic integrity, population structure, and genetic diversity in *Saccharum* germplasm. We identified a high rate of clone mislabeling and redundancy within the sugarcane germplasm collection, characterized by clone duplicate errors, homonymous off-types, and mistakes in species memberships. Moreover, our analyses of population structure and genetic diversity revealed novel insights into the classification and inter-relationships of *Saccharum* species. Overall, these findings provide valuable information for sugarcane research community to improve the accuracy and efficiency of managing and utilizing the sugarcane genetic resources in the WCSRG.

## 2 Materials and methods

### 2.1 Plant materials

**Figure 1:**
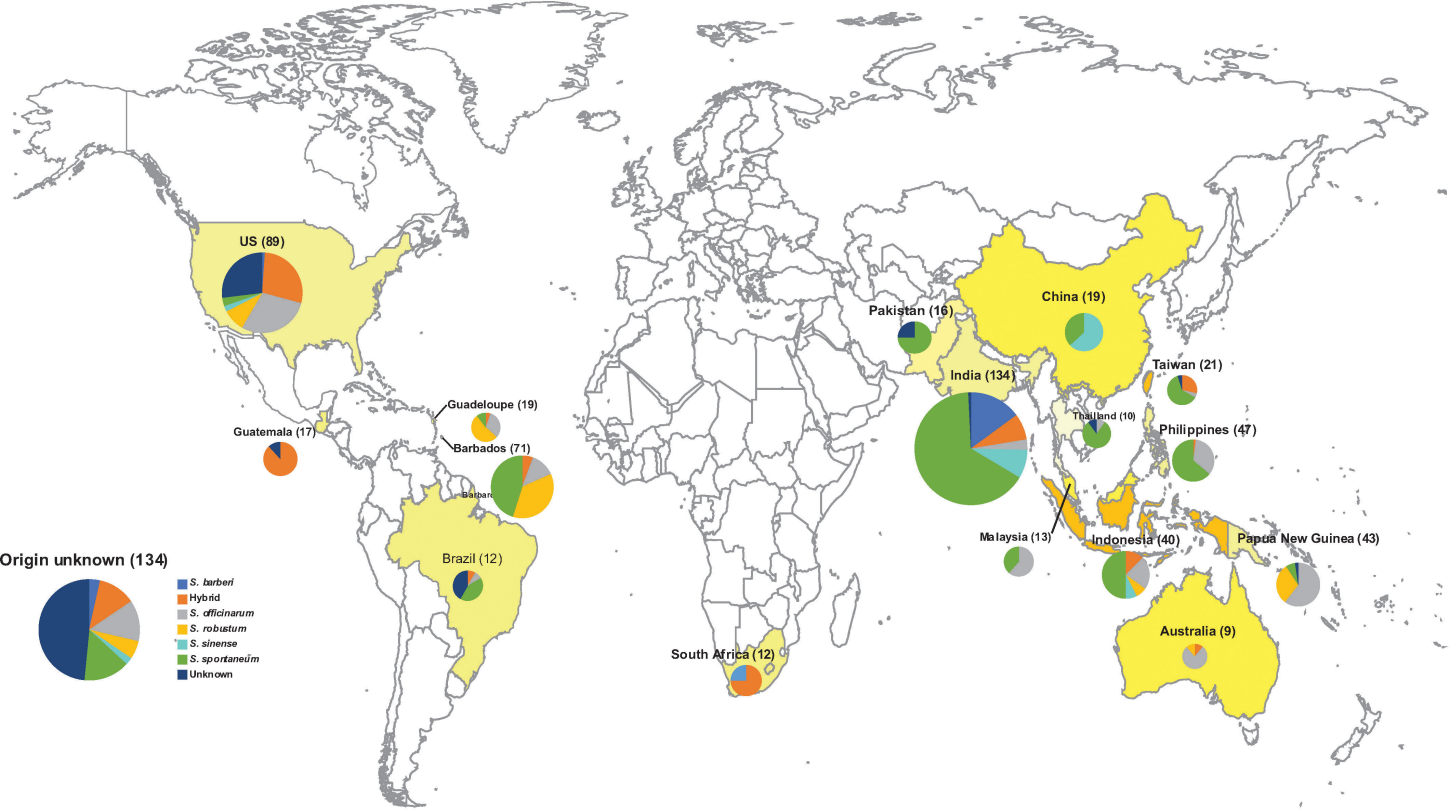
The Geographical origin of 901 *Saccharum* germplasm accessions analyzed in the present study, including *S. spontaneum* (283), *S. robustum* (76), *S. officinarum* (158), *S. barberi* (32), *S. sinense* (46), *S. edule* (3), *Saccharum* spp. (175), Unknown (3), hybrid (123). A detailed list with passport information is presented in **Table S1**.

The sugarcane accessions reported in this study are part of the WCSRG, which is curated by the USDA-ARS, SHRS, in Miami, FL. **Figure 1** provides a summary of the geographical distribution of the germplasm accessions. A detailed list of all accessions is provided in **Table S1**. The S. spontaneum accessions are maintained in 7-gallon pots on a concrete pad and not allowed to flower as they are considered invasive. The rest of the accessions are planted in the field and rotated to new field plots every 4 years. The mature plants are cut to the ground every year in the early spring until replanting. The species name of each accession in the WCSRG was defined based on the curator’s naming system.

From each sugarcane plant, one fully expanded young leaf was collected into labeled paper envelopes. A total of eight leaf disks were collected using the BioArk Leaf kit provided by LGC, Biosearch Technologies (https://www.biosearchtech.com/). The prepared BioArk Leaf kits were then submitted to LGC Genomics (Middleton, WI) for DNA extraction and subsequent genotyping.

### 2.2 SNP markers, Genotyping and SNP calling

The single-dose markers were initially selected from the Axiom Sugarcane 100K SNP array, which was developed based on five *Saccharum* species (*S. sinense*, *S. barberi*, *S. robustum*, *S. officinarum*, and *S. spontaneum*) and 37 sugarcane hybrids (You et al., 2019). A set of two thousand bi-allelic and single-dose SNPs were randomly selected from the Axiom Sugarcane 100K SNP array. Probes targeting these SNPs were designed by Biosearch Technologies (https://www.biosearchtech.com) and used to amplify sugarcane genome DNA libraries, which were then sequenced using the 1x 75 bp Illumina sequencing platform. Reads were trimmed by removing the first 40 bases and quality-checked with a Q value >20. The 2000 candidate SNPs were first evaluated in a pilot study using 196 sugarcane germplasm accessions (**Table S1**). Based on call rate, Minor Allele Frequency (MAF), and Linkage Disequilibrium (LD), we then selected a low-density genotyping panel including 400 SNPs and used it to genotype all the *Saccharum* clones including six *Saccharum* species and inter-specific hybrids.

The genotyping was performed using a targeted genotyping-by-sequencing approach called SeqSNP, which has been successfully used in several crops (Zhang et al., 2020; Jo et al., 2021; Ziarsolo et al., 2021). Sequence reads were aligned to the sequences (300bp) flanking the SNP markers, using Bowtie2 (version 2.4.5) (Langmead and Salzberg, 2012). The subsequent alignments (BAM files) were used to call SNP variants by the freebayes program (version 1.1.0) (Garrison and Marth, 2012) with the following parameters: min-quality 20, –min-supporting-allele-qsum 10; –read-mismatch-limit 4; –mismatch-base-quality-threshold 10; –exclude-unobserved-genotypes; –no-mnps; –no-complex; –ploidy 4; –min-alternate-fraction.08333; –legacy-gls. The called SNP variants (VCF files) were further filtered using the following criteria: 1) SNPs must be biallelic; 2) SNPs must be supported by at least 20 reads, otherwise, they were marked as missing genotypes; 3) both alternative and reference alleles must each be supported by at least 2 reads. After the initial filtering, informative SNPs were selected by excluding SNPs with a missing rate of 10% and a minor allele frequency of <5%. Additionally, samples with 10% or more missing SNP genotyping were also excluded. These filtering processes resulted in a dataset of 357 SNPs and 901 samples for downstream analyses. To assess the degree of variation among SNP markers, genetic parameters such as minor allele frequency (MAF), expected heterozygosity (H_exp_), and observed heterozygosity (H_obs_) for each SNP marker were measured using the R-package snpReady (version 0.9.6) (Granato et al., 2018).

### 2.3 Clone mislabeling and genetic redundancy

For this study, we defined three types of problems related to genetic integrity of sugarcane germplasm. The first type was synonymous mislabeling or “duplicate error,” meaning that sugarcane clones had different names but shared the same SNP genotype. The second was homonymous mislabeling, meaning that individual clones had the same name in this collection, but they had different SNP genotypes. The third type was mistakes, inaccuracies, or a lack of information in species classification, where the species membership of a given clone was wrongfully recorded (Brown et al., 2007; Yang et al., 2019).

To identify synonymous mislabeling among sugarcane clones, the allele difference between each pair of individuals was computed using the R-package poppr (Kamvar et al., 2014). Individuals with zero allele difference at all loci were considered duplicates. The groups of duplicates were visually inspected by constructing a network graph using the R-packages of network and ggplot2. Since genotyping errors are not uncommon, a pair of clones that differ by one or two loci could be the same clone (Kalinowski et al., 2006; Zhang et al., 2006). To assess potential genotyping error, we included 12 sugarcane samples in genotyping. These 12 samples were propagated from a single clone “P-Mag-84-2” (**Table S1**) and served as an internal control. Mismatched SNP loci among these 12 samples were calculated and used as a baseline to determine the “mismatch threshold” for clone identification. Any pair of samples that had mismatched loci below the threshold (near-identical genotypes) were considered as putative duplicates (Zhang et al., 2006; Akpertey et al., 2021).

The statistical rigor of duplicate identification was assessed using the probability of identity that two individuals may share the same multilocus genotype by chance (Waits et al., 2001). The computer program GenAlEx 6.5 (Peakall and Smouse, 2006; Peakall and Smouse, 2012) was used to calculate the probability of identity among siblings (PIDsib). PIDsib is defined as the probability that two sibling individuals drawn at random from a population have the same mutilocus genotype (Waits et al., 2001).

To identify homonymous mislabeling, SNP genotypes of the clones with the same name were manually grouped and compared using multi-locus matching. If the clones differ by more than two loci, then these clones were considered to have different SNP genotypes thus were claimed as homonymous mislabeling.

To identify clones with mistake in species membership, assignment test based on Bayesian clustering analysis of population structure (Pritchard et al., 2000) was used. Clones with wrongfully assigned species membership were detected and corrected based on the assignment result (see the next section).

### 2.4 Population structure and genetic diversity

To assess population structure in the collection, we only used accessions with explicit species names recorded in the passport data. Clones recorded as ‘Hybrid’ or ‘Unknown’ were excluded in this stage, which led to the retention of 591clones for the population structure analysis. The computer program STRUCTURE ver. 2.3.4 (Pritchard et al., 2000) was used. The program was run at 10 independent repetitions for population numbers ranging from K = 1 to K = 10, with a burn-in period of 50,000 and 100,000 Markov chain Monte Carlo (MCMC). The optimal number of model components (K) was determined based on delta K (Evanno et al., 2005). Based on the result, iterative runs were performed on each partitioned cluster to explore the sub-structures within each cluster, as recommended by Evanno et al. (2005). Ancestry and admixture proportions were visualized using computer program CLUMPAK (Kopelman et al., 2015).

Based on the result of the STRUCTURE analysis, clones with the assignment coefficient above 0.80 (Q value >0.80) were considered core members of each cluster and retained for subsequent genetic diversity analysis, including computation of F statistics, Analysis of Molecular Variance (AMOVA), Principal Coordinate Analysis and Neighbor-Joining Clustering Analysis.

AMOVA was performed using the program GenAlex 6.5 (Peakall and Smouse, 2006; Peakall and Smouse, 2012). The significance of fixation index (F_ST_) was tested using 999 random permutations. In addition, the F_ST_ for each pair of core germplasm groups was calculated and the statistical significance was tested using permutations with the program GenAlEx 6.5.

Key summary statistics including gene diversity (H_exp_) and observed heterozygosity (H_obs_) were calculated for each species using the program GenAlEx 6.5 (Peakall and Smouse, 2006; Peakall and Smouse, 2012). To illustrate genetic relationships among the species, a distance-based multivariate analysis was performed. Pairwise genetic distances were computed using the Distance option, and Principal Coordinates Analysis (PCoA) within the GenAlEx 6.5 program. The PCoA results are presented as two-axis PCO plots, and both plots axis 1 vs 2 and axis 1 vs 3 are presented separately.

To further examine the genetic relationship among different species, a neighbor-joining (NJ) clustering analysis was performed. The NJ tree was constructed based on the SNP genotype data using the R-package poppr (Kamvar et al., 2014). Pairwise genetic distances between the sugarcane clones were estimated and the neighbor-joining method was used to construct the tree. The final tree was visualized using FigTree version1.4.4 (http://tree.bio.ed.ac.uk/software/figtree).

To assess species membership and parentage for clones recorded as ‘hybrid’ or ‘unknown’, we used the core members of the five *Saccharum* species as references and increased their samples size to 500 for each species, using the Simulation procedure implemented in the computer program ONCOR (https://www.montana.edu/kalinowski/software/oncor.html). The simulated populations were then analyzed together with the 298 clones with unclarified species membership (clones labeled as ‘unknown’ or ‘hybrid’) using STRUCTURE 2.3.4. An admixed model was selected, and the number of clusters (K value) was set to five, corresponding to the five *Saccharum* species. Ten independent runs were conducted at K = 5, each consisting of 100,000 iterations after a burn-in period of 50,000 iterations. From the 10 independent runs, the mean membership score was presented as the inferred species/parentage or species membership.

## 3 Results

### 3.1 Genotyping with SNP markers

After applying an initial filtering process to exclude SNPs and samples with a missing rate of 10% or greater, a total of 751 SNP markers and 901 clones were obtained. Among the *Saccharum* clones, there were 286 *S. spontaneum*, 175 unknown, 158 *S. officinarum*, 123 hybrids, 76 *S. robustum*, 46 *S. sinense*, 32 *S. barberi*, 3 *S. edule*, 1 *S. narenga*, 1 *S. arundinaceum*, as recorded in passport data (**Table S1**). Of the SNPs, 217 (29%) were found to be monomorphic. To obtain informative SNP markers, we further filtered out SNPs with a minor allele frequency of less than 5%, resulting in a final set of 357 markers. The final genotype data for these markers showed an average missing rate of 0.15%, ranging from 0 to 7.6%. The sugarcane clones, on the other hand, showed an average missing rate of 0.15%, ranging from 0 to 6.2%. Among the 357 SNP markers, the expected heterozygosity ranged from 0.09 to 0.5, with an average of 0.23. The observed heterozygosity ranged from 0.09 to 0.99 with an average of 0.3. Additionally, the minor allele frequency ranged from 0.05 to 0.5 with an average of 0.153 (**Table S2**).

### 3.2 Clone mislabeling and genetic redundancy in the collection

**Figure 2:**
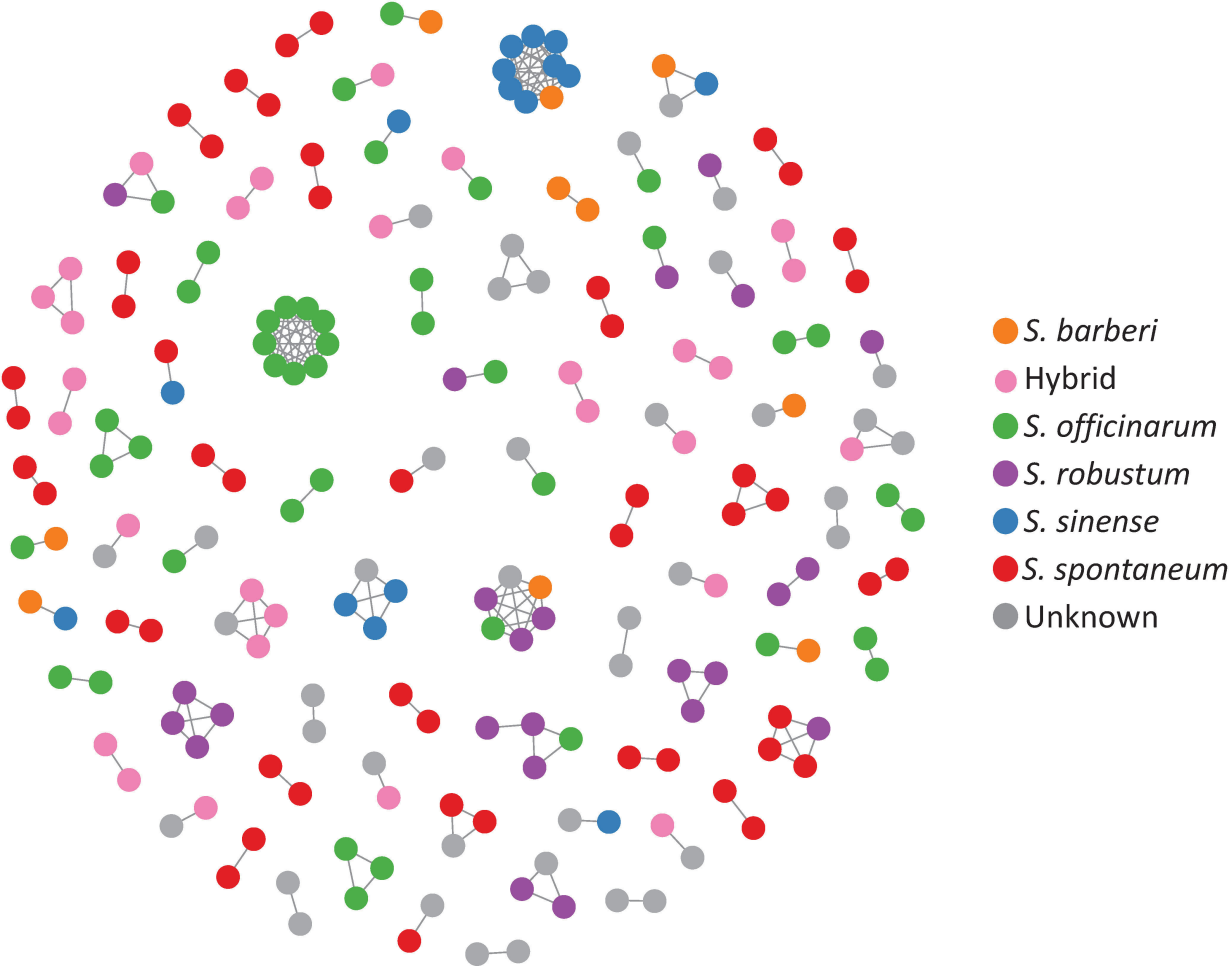
A network representing the genetic relationships among sugarcane clones based on allele difference at the SNP loci. The network was constructed with clones with zero allele difference (identical genotype) where nodes represent clones, and the connections indicate identical genotype between the clones.

Clonal propagation and field maintenance of sugarcane germplasm plants can often lead to mislabeling and name loss. The WCSRG, which houses collections from diverse locations worldwide, often encounters duplicated accessions with different regional names but identical clones. To estimate mislabeling and clonal redundancy, we measured the allele difference distance between each pair of sugarcane accessions at all SNP loci. Synonymous groups (duplicates) were determined when clones shared the same alleles at all SNP loci. Our analysis revealed 86 groups of duplicates comprising 211 accessions (23% of the examined *Saccharum* clones), demonstrating a high rate of synonymous mislabeling and genetic redundancy within the collection (**Figure 2**). The number of duplicated clones within each group ranged from two to nine clones, with 67 groups consisting of two clones (**Table 1**; **Table S3**).

**Table 1:** Examples of the identified synonymous mislabeling groups (duplicates) in the *Saccharum* germplasm maintained in the WCSRG.

Group	Clone code	Species	Clone Name	Source of introduction
2	SAC0472	*S. sinense*	UBA NAQUIN	US
	SAC0513	*S. sinense*	NEPAL 3	Nepal
	SAC0518	*S. sinense*	CHINA	South Africa
	SAC0519	*S. sinense*	AGAUL	South Africa
	SAC0531	*S. sinense*	TANZHOU	china
	SAC0616	*S. sinense*	MCILKRUM	US
	SAC0670	*S. barberi*	Kinar	India
	SAC0711	*S. sinense*	Uba Striped	Unknown
	SAC0760	*S. sinense*	Cayana 10	Unknown
3	SAC0321	*S. robustum*	IN 84-045	Barbados
	SAC0425	*S. barberi*	MESANGEN	Guyana
	SAC0426	*S. robustum*	IN 84-045	Barbados
	SAC0458	*S. officinarum*	HORNE	Barbados
	SAC0461	*S. robustum*	NG 28-251	Guadeloupe
	SAC0476	unknown	UNKNOWN	Unknown
4	SAC0323	*S. officinarum*	NG 28-014 (SS 58-08)	Papua New Guinea
	SAC0436	*S. robustum*	NG 57-208	US
	SAC0447	*S. robustum*	NG 57-208	US
	SAC1234	*S. robustum*	NG 57-208	US

The full list of identified duplicates and near-identical genotypes (clones differing by one or two alleles) was listed in **Table S3** and **S4**.

To estimate genotyping error, twelve clonal samples propagated from a single clone of ‘P-Mag-84-2’ were included as an internal control. Of the 12 samples, however, only eight samples (Group1 in the **Table S3**) were identified as duplicates with zero allele difference, while three samples showed one allele difference, and one sample showed two allele differences. These differences were attributed to likely genotyping errors at four loci. Assuming no mutation in the clonal plants, these results indicated an error rate of 0.093% in our genotyping, as four loci were called wrongly out of a total of 4,284 loci in the 12 clonal samples. Based on the observed error rate, we relaxed the threshold of detecting duplicates. Any pair of samples that had up to two allele differences were considered putative duplicates. Based on this relaxed threshold, we identified 135 groups consisting of 471 clones (52% of the total clones) as putative duplicates. The number of duplicated clones within each group ranged from 2 to 38 (**Table S4**).

The result of duplicate identification was supported by the probability of identity among siblings (PID-sib). The cumulative PID-sib of the first 48 SNPs ranged from 3.85E-04 (*S. robustum*) to 6.73E-07 (*S. officinarum*), which demonstrated that a high level of statistical power can be achieved in sugarcane germplasm analysis using only a small fraction of the 357 SNP markers (**Table S5**; **Figure S1**). When all 357 SNP loci were included, the cumulative PID-sib ranged from 2.4E-20 (*S. robustum*) to 7.6E-42 (*S. officinarum*), which indicates that there is almost a null probability of finding two individual clones with the same genotype within any of the five *Saccharum* species.

To identify homonymous mislabeling, clones with the same name were compared for their SNP genotypes using multi-locus matching. A total of 27 homonymous mislabeling groups were detected in all the studied *Saccharum* species, except *S. edule* (**Table 2**). Most of the identified homonymous groups were collected from the same country and geographical region, indicating that mislabeling occurred before the germplasm were introduced into WCSRG.

**Table 2:** Identified homonymous mislabeling groups in the *Saccharum* germplasm maintained in the WCSRG.

Homonymous mislabeling group	Name	Code	Species	Origin
1	AGOULE	SAC0451	*S. sinense*	India Tamil Nadu
2	BA 11569	SAC0638	*S. officinarum*	Barbados
3	Chino	SAC0577	*S. officinarum*	Hawaii
4	CO 312	SAC0636	Hybrid	South Africa
5	CO 313	SAC0635	Hybrid	South Africa
6	CP 01-1372	SAC0552	Unknown	Florida
7	CP 91-555	SAC0653	Unknown	Louisiana
8	F 154	SAC0605	Hybrid	Taiwan
9	F31-762	SAC0608	Unknown	Hawaii
10	F36-819	SAC0299	*S. officinarum*	Hawaii
11	HC 71	SAC0511	*S. officinarum*	Hawaii
12	IJ 76-414	SAC0460	*S. robustum*	Barbados
13	IJ 76-478	SAC0659	*S. officinarum*	Indonesia
14	IJ 76-480	SAC0602	*S. robustum*	Barbados
15	IJ 76-547	SAC0322	*S. robustum*	Guadeloupe
16	IN 81-014	SAC0328	*S. robustum*	Barbados
17	Kerah	SAC0515	*S. sinense*	Indonesia Java
18	Longchuan (Yunan)	SAC1228	*S. spontaneum*	China
19	MESANGEN	SAC0514	*S. barberi*	Guyana
20	MOL 6077	SAC0481	*S. robustum*	Hawaii
21	MOL 6427	SAC0569	Unknown	Hawaii
22	N 26-14	SAC0522	Unknown	Unknown
23	NG 57-208	SAC1234	*S. robustum*	Hawaii
24	NG 57-238	SAC0598	*S. robustum*	Barbados
25	NG 77-094	SAC0396	*S. robustum*	Papua New Guinea
26	SES 519	SAC218	*S. spontaneum*	India
27	Tanzhou	SAC0646	*S. sinense*	China Guangxi

Each of the 27 accessions have at least one homonymous accession with different SNP genotype.

### 3.3 Population structure and genetic diversity

#### 3.3.1 Bayesian clustering analysis

**Figure 3:**
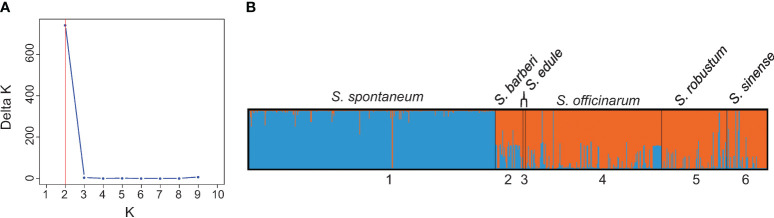
**(A)** Number of clusters based on the Evanno’s Delta K value. **(B)** Population structure of the 591 *Saccharum* germplasm accessions with explicit passport record of species memberships partitioned using Structure v2.3.4. Black vertical lines indicate the separation of the *Saccharum* species. Multiple colors within the genetic group imply admixed individuals under the scenario of K=2.

The results of population structure analysis on 591 clones (with explicit passport records of species names) are presented in **Figure 3**. According to the delta K method (Evanno et al., 2005), the most probable number of genetically distinct groups (K) was estimated to be two (**Figure 3A**). At K = 2, the *S. spontaneum* clones were clearly classified as a distinct cluster, while the other five species were assigned to the second cluster (shown in orange in **Figure 3B**). It’s noticeable that majority of the *S. sinense* clones had full population membership of the second cluster (in orange), whereas the majority of the *S. barberi* clones showed admixed genotypes between the first cluster (in blue) and the second cluster (in orange).

**Figure 4:**
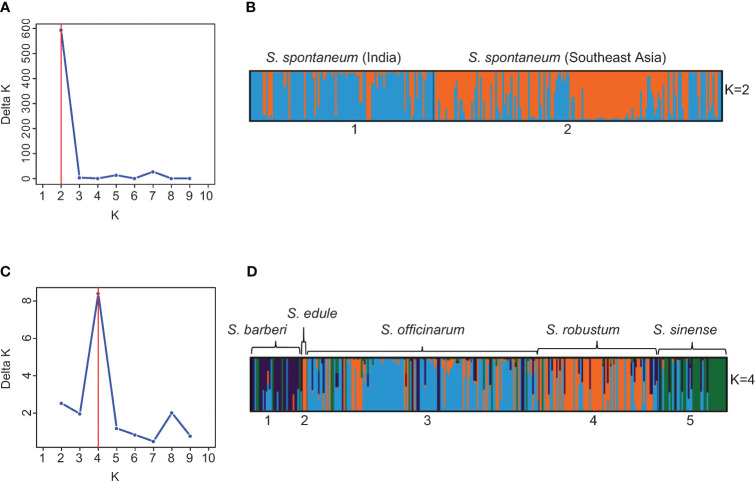
**(A)** Number of clusters in the 273 *S. spontaneum* clones based on the Evanno’s Delta K value. **(B)** Partitioned result of the 273 *S. spontaneum* clones at K = 2 using Structure v2.3.4. **(C)** Number of clusters in 299 clones of *S. barberi, S. officinarum, S. robustum/S. edule*, and *S. sinense* based on the Evanno’s Delta K value. **(D)** Population structure of the 299 clones of *S. barberi, S. officinarum, S. robustum/S. edule*, and *S. sinense* obtained using Structure v2.3.4. Multiple colors within the genetic group imply admixed individuals.

To further explore the substructure in the two clusters (*S. spontaneum* vs. the rest species), we repeated STRUCTURE analysis on each cluster using the same procedure and parameters. Through hierarchical analysis, the most probable number of genetically distinct groups (K) was two in the *S. spontaneum* cluster and four in the rest of the species (**Figures 4A, C**), based on Evanno’s Delta K method. Therefore, the hierarchical partitioning classified the *Saccharum* germplasm into six sub-clusters: the *S. spontaneum* clones were classified into two populations, of which the first population was mainly originated from India and nearby countries in South Asia, whereas the second population were mainly originated from Southeast Asia and Barbados. Hereinafter, we used *S. spontaneum* (India) and *S. spontaneum* (SE Asia) to represent these two populations in subsequent analyses. The other four clusters correspond to four distinguishable species including 1) *S. barberi*, 2) *S. officinarum*, 3) *S. robustum*/*S. edule*, and 4) *S. sinense* (**Figure 4D**; **Table S6**).

This result is highly compatible with the current taxonomy framework of *Saccharum* (*sensu stricto*), which proposes six *Saccharum* species, with *S. edule* considered as a mutant of *S. robustum* (Daniels and Roach, 1987). Based on this result, we also observed that many clones had mislabeled species membership. In total, we detected 141 cases of mistakes or inaccuracies in species membership. The largest group was found in *S. officinarum* (65), followed by *S. robustum* (32), *S. sinense* (16) and *S. barberi* (12). In contrast, only one clone of *S. spontaneum* was found to have mislabeled species membership (“IN 84-072” from Indonesia), in addition to 10 clones of hybrids derived from *S. spontaneum* (**Figures 4B, D**; **Table S6**).

To further understand the genetic relationships among the five *Saccharum* species, we selected the core members of each species, based on the membership coefficient (Q-value) generated by the STRUCTURE analysis, with the threshold ≥ 0.80 (**Table S6**). This stringent cutoff enabled the inclusion of clones with minimal admixture. A total of 412 core members with unique genotypes were retained and each clone had a unique SNP genotype. These 412 core members were used in subsequent analysis of genetic diversity, including PCoA, Neighbor-Joining clustering analyses and AMOVA.

#### 3.3.2 Principal Coordinates Analysis

**Figure 5:**
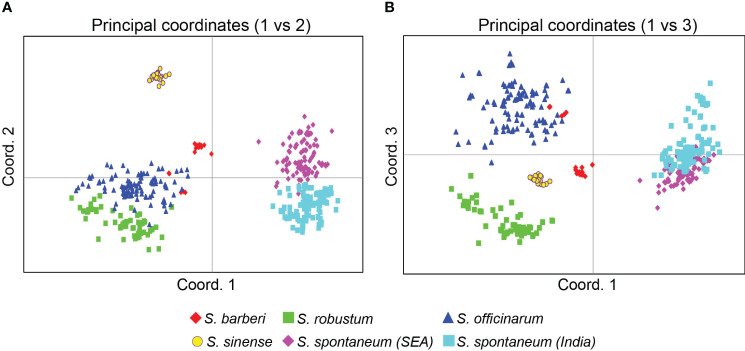
PCoA plots of the five *Saccharum* species represented by 412 core members with unique genotypes. **(A)** coordinates 1 vs 2 and **(B)** coordinates 2 vs 3. The analysis includes clones classified by STRUCTURE analysis with Q value >0.80. Each point represents an individual clone. The first three main axes accounted for the following percentages of the total variation: first axis = 21.7%, second axis = 13.0% and third axis = 8.0%.

The PCoA based on the Euclidean distance provided additional information on the relationships among the *Saccharum* germplasm clones (**Figure 5**). The first three principal coordinates accounted for 42.7% of the total variation, with the first, second and third coordinates explaining 21.7%, 13.0%, and 8.0%, respectively. Consistent with the findings from the STRUCTURE analysis, the core members of the five species were clearly separated from each other in both **Figures 5A, B**.

#### 3.3.3 Neighbor-Joining clustering analysis

**Figure 6:**
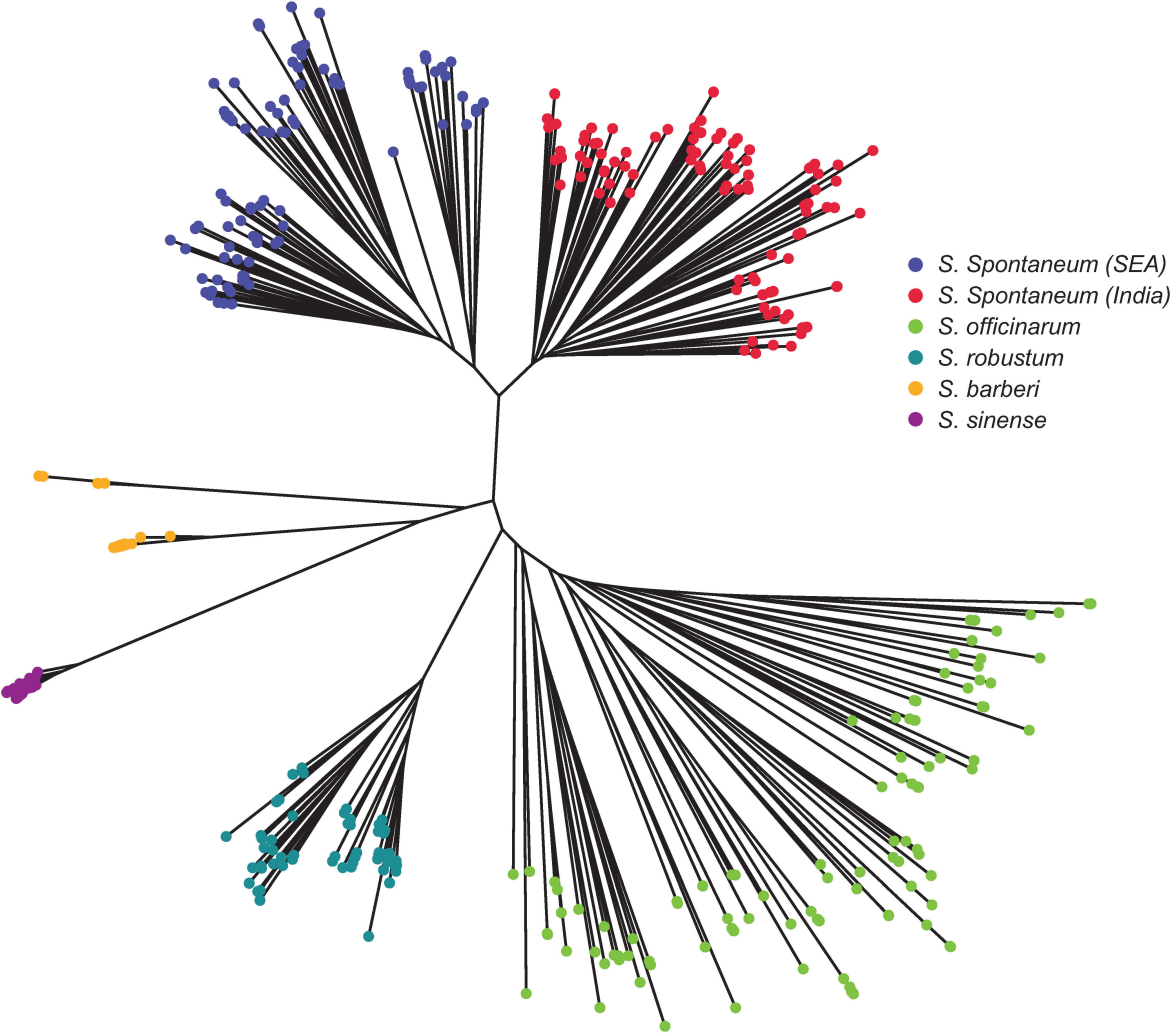
Neighbor-Joining tree depicting the relationships among the five *Saccharum* species represented by 412 core members with unique genotypes. The species *S. spontaneum* included two populations from India and Southeast Asia.

The Neighbor-Joining tree (**Figure 6**) revealed consistent results with STRUCTURE (**Figure 4C**) and PCoA analyses (**Figure 5**). There were two main clusters in the core members of the five species. Cluster 1 consisted of *S. officinarum*, *S. robustum*, *S. barberi*, and *S. sinense*, while Cluster 2 included the two populations of *S. spontaneum* from India and Southeast Asia. Within Cluster 1, *S. officinarum* and *S. robustum* were grouped together, showing their close relationship (**Figure 6**).

#### 3.3.4 F statistics and Analysis of Molecular variance

The pattern of genetic differentiation between the five sugarcane species was also reflected by pairwise F_ST_ values, where higher F_ST_ values indicate greater genetic differentiation (Weir and Hill, 2002). The lowest pairwise F_ST_ values (0.073) was found between the two *S. spontaneum* populations (India vs. Southeast Asia), indicating a low level of differentiation. Among the five species, the pairwise F_ST_ ranged from 0.103 (*S. officinarum vs S. barberi*) to 0.323 (*S. robustum* vs *S. sinense*) (**Table 3**). The pairwise F_ST_ values generally align with the results from the PCoA plot (**Figure 5**) and the phylogenetic tree (**Figure 6**). All F_ST_ were highly significant (P < 0.001) based on permutation test. However, it’s noteworthy that *S. sinense* was found to have the highest mean F_ST_ value (0.239), followed by *S. robustum* (0.224), *S. barberi* (0.178), *S. spontaneum* (0.176) and *S. officinarum* (0.123).

**Table 3:** Pairwise population F_ST_ analysis among the five *Saccharum* species, represented by 412 core members with unique genotypes.

Species	*S. barberi*	*S. robustum*	*S. officinarum*	*S. sinense*	*S. spontaneum* (SE Asia)	*S. spontaneum* (India)
*S. barberi*	0.000	0.248	0.103	0.204	0.161	0.176
*S. robustum*	0.248	0.000	0.107	0.323	0.218	0.224
*S. officinarum*	0.103	0.107	0.000	0.168	0.119	0.120
*S. sinense*	0.204	0.323	0.168	0.000	0.216	0.286
*S. spontaneum* (SE Asia)	0.161	0.218	0.119	0.216	0.000	0.073
*S. spontaneum* (India)	0.176	0.224	0.120	0.286	0.073	0.000
	0.178	0.224	0.123	0.239	0.157	0.176

The probability, P (rand >= data) based on 999 permutations is shown above the diagonal.

AMOVA was employed to assess the distribution of the observed genetic variance among the five sugarcane species, excluding hybrids and unknown species. The results showed that a substantial proportion of the total genetic variation (85%) was attributed to within-species variation, while the remaining 15% of the total genetic variance was found in variation between species (**Table 4**). This shows that the observed genetic variations primarily arise from variation among individual clones within species rather than between different species. Out of the five species, *S. officinarum* has the highest intra-specific molecular variance (46.1), indicating its status as a cultivated hybrid species with extensive gene introgressions. In contrast, *S. robustum* had the lowest intra-specific molecular variance (21.8), suggesting its status as an ancient species with limited inter-specific gene flow. Intra-specific molecular variance in *S. spontaneum*, *S. barberi* and *S. sinense* are comparable. It’s noticeable that within *S. spontaneum*, the population from Southeast Asia had higher molecular variance (35.0) than the population from India (30.8).

**Table 4:** AMOVA and partitioning of total molecular variance within and among the five *Saccharum* species represented by 412 core members with unique genotypes.

Source	df	SS	MS	Est. Var.	%	P value
Among Pops	5	4310.9	862.2	6.4	15%	<0.001
Within Pops	818	28509.8	34.9	34.9	85%	
*S. barberi*	37	1257.5	34.0			
*S. robustum*	123	2682.6	21.8			
*S. officinarum*	207	9543.0	46.1			
*S. sinense*	55	2000.1	36.4			
*S. spontaneum* (S.E. Asia)	197	6898.5	35.0			
*S. spontaneum* (India)	199	6128.1	30.8			
Total	823	32820.7		41.2	100%	

*Probability, P (rand >= data), for F_ST_ is based on standard permutation across the full data set.

#### 3.3.5 Observed heterozygosity and genetic diversity

*S. spontaneum* and *S. robustum* are considered as wild sugarcane species (Dinesh Babu et al., 2022). It is interesting to find that these two species exhibited relatively lower gene diversity and higher homogeneity compared to other cultivated species (**Table 5**). Notably, Zhang et al. (2018) also reported low gene diversity across 64 *S. spontaneum* accessions, based on genome sequencing data. They showed that the nucleotide diversity was much lower than that of other clonally propagated crops such as potato, cassava, grape, and citrus. Modern sugarcane cultivars have been extensively developed through interspecific crosses between *S. spontaneum* and *S. officinarum*. This disparity between wild and cultivated accessions suggests that the lower gene diversity observed in *S. spontaneum* is a characteristic of natural populations without human intervention. In contrast, the extensive intercrossing between species has likely contributed to the increased gene diversity and heterozygosity in hybrid cultivars.

**Table 5:** Sample size (N), Observed heterozygosity (H_obs_) and Gene diversity (H_exp_) in the five *Saccharum* species represented by 412 core members.

Species	N	H_obs_		H_exp_	
		Mean	SE	Mean	SE
*S. barberi*	19	0.302	0.020	0.183	0.011
*S. robustum*	62	0.162	0.014	0.114	0.009
*S. officinarum*	104	0.354	0.014	0.256	0.008
*S. sinense*	28	0.389	0.025	0.199	0.013
*S. spontaneum (SEA)*	99	0.285	0.017	0.193	0.010
*S. spontaneum (India)*	100	0.238	0.015	0.169	0.009

### 3.4 Inferred species membership and parentage for clones with missing passport information

Of the 901 *Saccharum* (*sensu stricto*) accessions maintained in this sugarcane germplasm collection, there were 175 clones that do not have clear passport data for their species membership. These clones were recorded as ‘unknown’ in the collection. In addition, there were 123 clones recorded as ‘Hybrid’, but their parentage information was lacking. Using the selected core members of the five *Saccharum* species as references, we were able to assign the species membership and parentage for all the clones that have ambiguous passport records. Our result showed that most of these clones have a species membership of *S. officinarum* (**Table S8**). Among the 175 clones recorded as ‘unknown’, two were assigned to *S. barberi*, 12 to *S. sinense*, 12 to *S. spontaneum*, 10 to *S. robustum*, 91 to *S. officinarum*, and 40 to inter-specific hybrids. Similarly, among the 123 clones that were recorded as ‘Hybrid’, two were assigned to *S. spontaneum*, one to *S. robustum*, 96 to *S. officinarum*, and 24 to inter-specific hybrid (**Table S9**).

## 4 Discussion

Genetic integrity is crucial for efficient conservation and use of plant germplasm for genetic improvement. This is particularly the case for many tropical/subtropical crops such as sugarcane, which is typically maintained in the field and propagated clonally, a process that can often result in mislabeling or loss of identifiers . Moreover, complex hybridization among sugarcane species further complicates the germplasm identification and highlights the need for comprehensive molecular and morphological characterization. However, accurate identification of sugarcane germplasm has been technically challenging, due to the high polyploidy (and aneuploidy) nature of this crop. In this study, we selected single-dose SNP markers and employed a targeted genotyping-by-sequencing (Seq-SNP) method to assess clone identity, population structure and genetic diversity in the *Saccharum* germplasm maintained in the WCSRG. This approach was chosen for its compatibility with sugarcane’s diverse ploidy levels and large genome. The single-dose SNP markers offers advantages for polyploid plants such as sugarcane by enabling the identification and differentiation of alleles present across multiple sets of chromosomes without the need of ploidy determination (Sorrells, 1992; Aitken, 2022). By employing this high-throughput genotyping technique, we significantly improved our understanding of the genetic integrity and species relationship of sugarcane germplasm. The result provided valuable information to ensure the accuracy and efficiency in managing the sugarcane collection and facilitate its utilization in breeding programs.

### 4.1 Clone mislabeling and genetic redundancy

Using the selected SNP markers, we genotyped 901 *Saccharum* clones from six species including hybrids. Based on the SNP genotypes, we discovered a high rate of clone mislabeling and genetic redundancy within the studied sugarcane collection. Because genotyping errors frequently occur, a pair of clones with a small number of mismatched loci could be duplicates as well (Zhang et al., 2006). Therefore, a threshold of mismatches to determine duplicates needs to be established. To evaluate the genotyping error rate, we included 12 samples propagated from the same clone as an internal control. Among the 12 samples, eight samples exhibited zero allele difference, while three samples showed one allele difference, and one sample showed two allele differences, suggesting that a two-allele difference could be used as the threshold for duplicate identification. Based on this threshold, we found that that half of the clones had at least one other clone with up to a two-allele difference (**Table S4**). We assessed how many SNP markers are needed to provide sufficient statistical power for sugarcane duplicate identification. Based on the cumulative PID-sib values for each species, we demonstrated that when utilizing 48 SNPs, the probability that two sibling individuals may share the same multilocus genotype by chance (Waits et al., 2001) was smaller than 0.001 (PIDsib <0.001) (**Table S8**; **Figure S1**). Therefore, the panel of 357 SNPs is far more than sufficient to identify synonymously mislabeled clones in each species (**Table S5**; **Figure S1**).

In addition to the detection of synonymous groups, the single-dose SNP genotyping enabled the identification of 27 homonymous mislabeling groups (**Table 2**), where clones with the same name had different SNP genotypes. These homonymous mislabeling groups were detected in all five *Saccharum* species, often in germplasm accessions collected from the same geographical region. For example, the three clones of “AGOULE” were all collected from Tamil Nadu, India. However, they exhibited two different SNP genotypes. In another case, two clones were labeled as “Chino”, both from Hawaii, but their SNP genotypes were different. A more noteworthy example is the two “Uba” clones from India. Although they were both classified correctly as *S. sinense*, they had different SNP genotypes. Since “Uba” has been widely used as an important source of disease resistance in sugarcane breeding, the identified homonymous mislabeling has significant implications on sugarcane genetic studies and new variety development.

Furthermore, a high rate of mislabeling and inaccuracies was also detected in recorded species membership. Most of the mislabeling and inaccuracies occurred in species pairs that shared morphological similarities, such as *S. officinarum* vs *S. robustum* and *S. barberi* vs *S. sinense*. In contrast, there was almost no mislabeling of species membership between *S. spontaneum* and the rest of species. The high rate of mislabeling revealed in the present study was likely due to sugarcane’s feature as a clonally propagated crop, which has allowed the exchange of sugarcane germplasm as clones among regions, countries, and continents. However, passport data, such as records and labels of the germplasm have not always followed the same naming conventions, leading to limited information about their correct identity. In fact, the majority of mislabeling and redundancy were observed in cultivated species, indicating a more intensive exchange of cultivated germplasm than wild species. Additional efforts of characterization are needed to fully resolve the mislabeling problem. SNP profiles for reference sugarcane clones need to be established through international collaboration. The putative mislabeled clones need to be compared with established references to correct the mislabeling errors. For the putative duplicate groups with near-identical genotypes, SNP genotyping will need to be repeated to confirm their clone identity. Moreover, since somaclonal mutation can occur in sugarcane, phenotypic examination remains essential to complement the result of molecular characterization.

### 4.2 Population structure and relationships among *saccharum* species

The sugarcane research community usually regarded *Saccharum* (*sensu stricto*) as containing six species, including two wild species (*S. spontaneum* and *S. robustum*), and four cultivated species - *S. officinarum*, *S. edule*, *S. barberi*, and *S. sinense* (Purseglove, 1972; Daniels and Roach, 1987; Grivet et al., 2006; Hemaprabha et al., 2022). The present study, using both model-based clustering and multivariant analysis based on 357 single dose SNP makers, supported the current classification of *Saccharum* germplasm. The only exception is *S. edule*, which could not be differentiated from *S. robustum*. *S. edule* is cultivated in New Guinea and nearby islands for its aborted edible inflorescences. Our result is consistent with the hypothesis that *S. edule* is a small group of sterile mutants that originated from *S. robustum* (Purseglove, 1972; Grivet et al., 2004; Grivet et al., 2006).

The present study also showed that *S. spontaneum* is a well-differentiated wild species, as shown by the analytical results of STRUCTURE (**Figure 3**), PCoA (**Figure 5**) and the NJ-tree (**Figure 6**). This observation is consistent with previous reports based on SSR markers (Brown et al., 2007), genome re-sequencing data (Yang et al., 2019; Fickett et al., 2020), and plastid genome sequences (Evans and Joshi, 2016). In the present study, we found very few mislabeling between *S. spontaneum* and the other four species. Nonetheless, the result of the hierarchical STRUCTURE analysis on *S. spontaneum* showed that there were two sub-groups within this species (**Figure 4A, B**). The first sub-group was mainly from India and south Asia, whereas the second sub-group was dominantly from Southeast Asia countries (**Table S6**). This result agrees with the recent report of Pompidor et al. (2021) and further indicates the importance of maintaining differentiated populations based on broader geographical regions.

A close genetic relationship was observed between *S. robustum* and *S. officinarum*. This observation is consistent with the recent finding of Pompidor et al. (2021), which suggested that both *S. officinarum* and *S. robustum* were derived from the same two ancestral genomes (A and B genomes), indicating a common origin of both species. Nonetheless, our result showed that the two species could be clearly differentiated at the molecular level, as demonstrated by the results of STRUCTURE (**Figure 4B**), PCoA (**Figure 5**) and NJ tree (**Figure 6**).

*S. barberi* and *S. sinense* are two cultivated species that are closely related (Purseglove, 1972; Lu et al., 1994; Hemaprabha et al., 2022). However, the taxonomy status, as well as the relationship between these two species, has been a subject of debate. It was proposed that *S. officinarum* hybridized with *S. spontaneum* in Asia continental and the hybrid progenies developed into *S. barberi* in India and *S. sinense* in China (Brandes, 1956; Daniels and Roach, 1987; D’Hont et al., 2002; Li et al., 2022). The geographical barrier (Southern China for *C. sinensis* vs. Northern India for *C. barberi*) could played significant role in the genetic differentiation of these two species. Using target enrichment sequencing of 307 germplasm accessions from WCSRG, Yang et al. (2019) showed that *S. sinense* and *S. barberi* were different in terms of genome compositions and potential ancestor accessions. Our result showed that *S. barberi* had the closest relationship with *S. officinarum*, which supported the proposal that *S. barberi* is a hybrid of *S. officinarum* and *S. spontaneum*. However, the present result also showed that relative to *S. barberi*, *S. sinense* had a larger genetic differentiation from *S. officinarum* and *S. spontaneum* (**Table 3**). Nonetheless, the number of samples of *S. barberi* and *S. sinense* used in the present study is relatively small. A systematic collection of samples representing the full geographical range of these two species is needed for a comprehensive analysis of population structure and genetic diversity in these two species.

To elucidate the underlying patterns of genetic variation in the sugarcane population, we conducted AMOVA to partition the observed variation among sugarcane clones. According to the AMOVA results, a significant proportion of the observed genetic diversity was attributed to variation among individual clones, accounting for 85% of the total variation, while the remaining 15% of the variation was attributed to variation between species (**Table 4**). These results are consistent with previous studies (Nayak et al., 2014; Manechini et al., 2018; Fickett et al., 2020; Singh et al., 2020), indicating that the primary source of genetic diversity resides within species rather than between different species. The relatively low percentage of genetic variation between species suggests that there may be a considerable level of introgression and systematic crossing occurring between different sugarcane species, reflecting intercrossing nature among sugarcane species (Moore et al., 2013). In all species, the observed heterozygosity was higher than the expected heterozygosity, which suggests more intercrosses between isolated populations than the founders. The inter-specific gene flow in sugarcane is well-supported by historical accounts that, throughout the seventeenth, eighteenth, and nineteen centuries, there was an extensive exchange of varieties among the sugarcane planters worldwide (Warner, 1962). The development of improved cultivars involved frequent intercrossing between the species, which also likely contributed to the low genetic variation between species. This is supported by the observed heterozygosity exceeding Hardy-Weinberg expectations, suggesting a greater number of intercrosses than expected from the founders alone.

In conclusion, accurate germplasm identity is critical for efficient management of sugarcane germplasm. Using single-dose SNP markers, we genotyped all the *Saccharum* clones in WCSRG, maintained at USDA-ARS. The single-dose SNP genotypes enabled us to detect a high rate of mislabeling and genetic redundancy in this collection. In addition, an analysis of population structure using both ordination and model-based clustering, revealed five genetic groups in the *Saccharum* germplasm, corresponding to *S. barberi, S. robustum, S. officinarum, S. sinense, and S. spontaneum*. The pattern of genetic structure in the *Saccharum* gene pool suggested a high level of gene flow among sugarcane species and across different geographical regions, likely facilitated by human intervention, as evident from the lower genetic variation observed between species. This extensive germplasm exchange, predominantly as clonal material, may contribute to the mislabeling and redundancy observed within the sugarcane collections. Through comprehensive analyses of genetic identity, we were able to detect genetic redundancy in the collection. Furthermore, we assessed the ancestral species/populations among the *Saccharum* germplasm clones and ascertained the presence of core members in each species. Using these core members as references, we were able to correct mistakes and/or inaccuracy in species membership, as well as clarify the parentage for hybrid clones. The corrected mislabeling in species membership needs to be validated based on phenotypic characteristics. Our results ensured that the preserved clones in the WCSRG have distinct genetic makeup. This is the first time that a large germplasm collection of sugarcane—a complicated polyploidy crop - was systematically characterized in terms of clone integrity and genetic redundancy. The single-dose SNP markers developed by this study offer a powerful tool for characterizing sugarcane germplasm worldwide. These markers can also be potentially used for identifying chromosomes. The reported findings have important implications for sugarcane genebank management, germplasm exchange, and crop genetic improvement.

## Data availability statement

The genomic DNA sequencing data are accessible in the Sequence Read Archive (SRA) under bioproject ID PRJNA1051683 (http://www.ncbi.nlm.nih.gov/bioproject).

## Author contributions

SP: Formal analysis, Investigation, Methodology, Software, Validation, Writing – original draft, Writing – review & editing. DZ: Conceptualization, Formal analysis, Funding acquisition, Investigation, Methodology, Resources, Software, Supervision, Writing – original draft, Writing – review & editing. GA: Conceptualization, Data curation, Investigation, Methodology, Project administration, Resources, Supervision, Writing – original draft, Writing – review & editing.
