# Supplementary material for: Assessing the genetic integrity of sugarcane germplasm in the USDA-ARS National Plant Germplasm System collection using single-dose SNP markers
Source: Front Plant Sci. 2024 Jan 4;14:1337736. doi: 10.3389/fpls.2023.1337736 (PMC10794611; doi:10.3389/fpls.2023.1337736)
Supplement: Supplementary file 1 [file DataSheet_1.docx]

**Assessing the genetic integrity of sugarcane germplasm in the USDA-ARS National Plant Germplasm System Collection** **using single-dose SNP markers**

Sunchung Park^1^, Dapeng Zhang^1*^, Gul Shad Ali^2^^*^

^1^Sustainable Perennial Crops Laboratory, United States Department of Agriculture, Agriculture Research Service, Beltsville, MD, United States.

^2^Subtropical Horticulture Research Station, United States Department of Agriculture, Agriculture Research Service, Miami, FL, United States.

*** Correspondence:**Corresponding Author

Gul Shad Ali [Gul.Ali@usda.gov](mailto:Gul.Ali@usda.gov) and Dapeng Zhang [dapeng.zhang@usda.gov](mailto:dapeng.zhang@usda.gov)

# Supplementary Figures and Tables

## Supplementary Figures


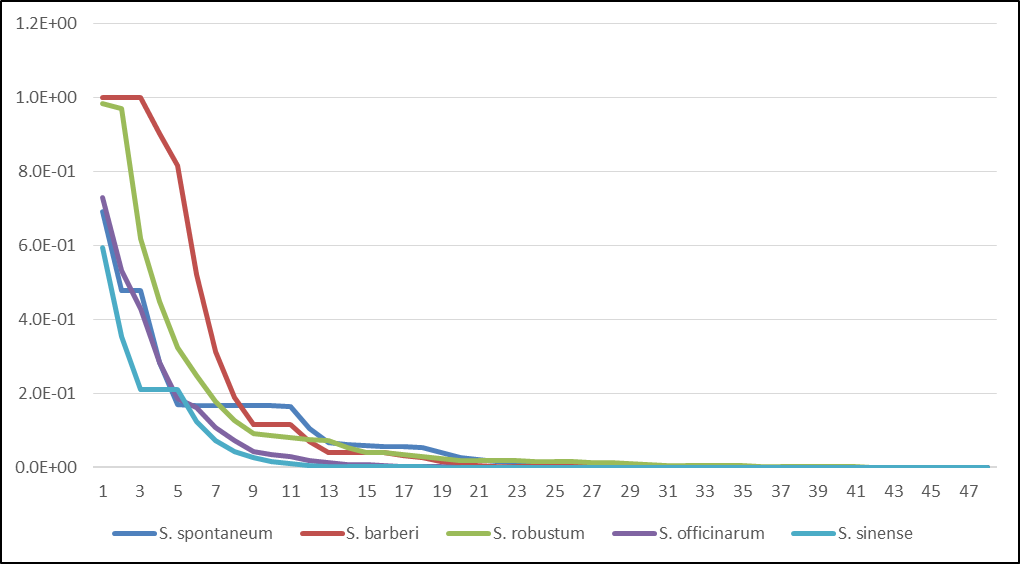


**Supplementary Figure 1.** The accumulated probability of identity among siblings (PID-sib) in five Saccharum species. PID-sib is defined as the probability that two sibling individuals drawn at random from a population have the same mutilocus genotype (Waits et al., 2001). The plot presented the accumulated PID_sibs on the first 48 SNPs only. The full result of accumulated PID_sibs for 357 SNPs was presented in Table S8. The y-axis represents PID_sibs. The x-axis shows the probability of finding two fully matched clones when 48 SNPs were genotyped. The colored lines demonstrated that PID_sibs in the five species approached zero (P<0.0001) when 48 SNPs were genotyped.
